# High Incidence of Hepatitis B Infection-Associated Cirrhosis and Hepatocellular Carcinoma in the Southeast Asian Patients with Portal Vein Thrombosis

**DOI:** 10.1186/1471-230X-11-66

**Published:** 2011-06-10

**Authors:** Korn Lertpipopmetha, Chirayu U Auewarakul

**Affiliations:** 1 Department of Medicine, Faculty of Medicine Siriraj Hospital, Mahidol University, Bangkok, Thailand

**Keywords:** *portal vein thrombosis*, *incidence*, *risk factors*, *cirrhosis*, *hepatocellular carcinoma*, *HBV infection*, *Southeast Asia*

## Abstract

**Background:**

Portal vein thrombosis (PVT) is a rare condition associated with serious morbidity and mortality. The objective of this study was to determine the frequency, clinical presentations, and risk factors of PVT from the set of data firstly collected among the Southeast Asian population.

**Methods:**

A retrospective study was undertaken to identify patients diagnosed with thrombosis of the portal system and other abdominal veins. The hospital medical records were retrieved based on the selected ICD-10 codes. Clinical presentations were collected and risk factors determined.

**Results:**

From 2000-2009, 467 hospital charts with designated ICD-10 codes of I81, I82.2, I82.3, I82.8, I82.9, or K55.0 were identified. PVT (I81) was the most common thrombosis (194 cases, 41.54%). The majority of PVT patients were males (65%), older than 40 years (75%), and presented with abdominal distension/ascites (69%), splenomegaly (54.6%), and abdominal pain (50.5%). Overall, the predominant risk factor was hepatocellular carcinoma (HCC) (52.5%), followed by liver cirrhosis without cancer (9.3%), abdominal infection/inflammation (9.3%), cholangiocarcinoma (8.2%), and abdominal intervention (7.7%). In young patients, abdominal interventions including umbilical catheterization (23.1%) and hepatectomy (7.7%) were the most frequent risks whereas in older cases, primary hepatobiliary cancer and cirrhosis (78%) were the major risks. Liver metastases from other organs were infrequently found. Chronic hepatitis B virus (HBV) infection was the main etiology associated with cirrhosis/HCC leading to PVT in this cohort. A third of the older PVT patients (age >40) had HBV and very few carried hepatitis C virus (HCV) whereas none of the young PVT patients (age <20) had HBV or HCV. A variety of abdominal infections/inflammations were also found including liver abscess, splenic abscess, cholangitis, cholecystitis, pancreatitis, omphalitis, and abdominal tuberculosis. Single cases of systemic lymphangiomatosis and Klippel-Trénaunay vascular malformation syndrome were also identified. Other thrombophilic conditions such as myeloproliferative neoplasms, paroxysmal nocturnal hemoglobinuria, protein S deficiency, and anti-phospholipid syndrome were rarely encountered.

**Conclusion:**

HBV is the major risk of PVT in the Southeast Asian population. Several risk factors identified in this population have rarely been described and some are remarkably different from those reported in the West. Host and environmental factors may play a causal role in the initiation and development of PVT in various ethnicities and geographic locations.

## 1. Background

Portal vein thrombosis (PVT) is considered a rare clinical and pathological entity [1,2]. Thrombus formation usually begins in the portal vein and sometimes extends to other branches of the portal system such as splenic vein or mesenteric vein [1,3,4]. The involved blood vessels can be partially or totally occluded resulting in increased portal venous outflow resistance from narrowing or obstructed blood vessel lumens [1,3,4]. As thrombosis usually occurs gradually giving time for the development of cavernous transformation as a result of periportal collateralization around the occluded portal vein, most patients are asymptomatic and do not seek medical attention [2-4]. However, obvious signs of portal hypertension such as splenomegaly, ascites, and massive hemorrhage from esophageal and gastric varices could also be found [1-6]. In the acute setting of moderate to severe thrombus occlusion, abdominal pain may be the striking presentation [2-4,6,7].

The prevalence of PVT is not well-defined due to its rarity and initial asymptomatic nature. Nevertheless, transient occlusion of the portal system is increasingly being recognized during the diagnostic course of various acute and chronic abdominal problems such as appendicitis, cholecystitis, cholangitis, pancreatitis, and other intra-abdominal infections by ultrasonography [6,7]. A proportion of patients with portal hypertension were also found coincidentally to have a thrombus in the portal system [1-4,6,8]. In a study of 701 hospitalized patients with cirrhosis who underwent routine ultrasound screening, PVT could be identified in 11% of the cases [3]. The largest series was a retrospective review of 23,796 autopsies which revealed the prevalence of PVT of 1% [1].

Since PVT is an uncommon and under-recognized disorder and its true prevalence is not presently established, it was of much interest to conduct a study to explore the frequency, clinical presentations, and risk factors of PVT at one of the largest hospitals in Southeast Asia. During a 10-year period, we were able to find 194 PVT cases and the majority of them presented with a classical sign of portal hypertension and advanced symptoms. Interestingly, the identifiable risk factors appeared to be mostly associated with hepatitis B virus (HBV) infection which is prevalent in this region. The fact that HBV-associated cirrhosis and hepatocellular carcinoma (HCC) are the predominant risks for PVT is strikingly different from the major risk factors in the Western reports. Other diseases that have rarely been described were also discovered in this series including Klippel-Trénaunay syndrome [9], systemic lymphangiomatosis [10], and abdominal tuberculosis [6].

## 2. Methods

This study was a retrospective analysis undertaken at Siriraj Hospital which is the largest hospital in Thailand comprising of more than 2,500 inpatient beds and more than 2 million outpatient visits a year. Patients diagnosed with thrombosis of the portal system and other abdominal veins from January 2000 to December 2009 were identified through the hospital's computerized medical records based upon the Tenth Revision of the International Statistical Classification of Diseases and Related Health Problems (ICD-10) coding system http://www.who.int/classifications/icd/en/. The study was approved by the Faculty of Medicine Siriraj Hospital Ethical Committee.

The following data were extracted from the medical records: demographic data (age and gender), clinical presentations (abdominal pain, abdominal distension, loss of appetite, nausea, vomiting, diarrhea, weight loss, splenomegaly, fever, jaundice, ascites, and gastrointestinal hemorrhage), complications (esophageal and gastric varices, variceal hemorrhage, portal hypertensive gastropathy, and ascites), extension of the thrombus (main, right or left branch of portal vein, mesenteric vein, splenic vein, vena cava), imaging methods used to diagnose PVT (ultrasound with or without Doppler, computed tomography (CT), magnetic resonance imaging (MRI) or magnetic resonance venography (MRV)), and hepatitis serology (HBV and hepatitis C virus (HCV)). Diagnosis of portal hypertensive gastropathy and grading of esophageal- and gastric varices was made by means of esophago-gastro-duodenoscopy (EGD). Owing to the severity of cancer and cirrhosis, the patients were classified into 2 groups: group 1) patients with cancer or cirrhosis and group 2) patients without cancer and cirrhosis. Patients were also categorized into 4 groups according to respective ages: group 1) < 20 years, group 2) ≥ 20 to 40 years, group 3) ≥40 to 60 years, and group 4) ≥60 years. Statistical analyses of continuous variables (mean, standard deviation (SD), and range) and categorical variables (number and percentage) were performed. A p-value was calculated when indicated.

## 3. Results

### 3.1 Incidence of PVT and other abdominal vein thrombosis

From 2000-2009, 467 hospital charts with designated ICD-10 codes of 181, I82.2, I82.3, I82.8, I82.9, or K55.0 were identified and extracted from the hospital system. PVT (I81) was the most commonly identified thrombosis (194 cases, 41.5%) among all abdominal venous thrombosis as shown in Table 1.

**Table 1:** Frequency of abdominal vein thrombosis

Thrombosis classified based on ICD-10 codes	No. of cases (%)
Portal vein thrombosis (I81)	194 (41.5)
Mesenteric vein thrombosis (K55.0)	18 (3.9)
Thrombosis of vena cava (I82.2)	81 (17.3)
Thrombosis of renal vein (I82.3)	18 (3.9)
Thrombosis of other specified vein (I82.8)	96 (20.6)
Thrombosis of unspecified vein (I82.9) Thrombosis of isolated splenic vein	52 (11.1) 3 (0.6)
Thrombosis of vena cava + renal vein	3 (0.6)
Thrombosis of vena cava + other specified veins	1 (0.2)
Thrombosis of vena cava + renal vein + other specified veins	1 (0.2)
**Total cases in this study**	**467 (100)**

Table 2 delineates the distribution of thrombosis in the portal system in the 194 patients diagnosed with PVT. Mainly, the portal vein was solely involved (145 cases, 74.7%). Other branches were less frequently involved including splenic vein, superior mesenteric vein, inferior mesenteric vein, and vena cava. The first diagnosis of PVT was established by ultrasonography (33 cases, 17%), Doppler ultrasonography (13 cases, 6.7%), CT (139 cases, 71.6%), MRI (2 cases, 1%), and MRV (7 cases, 3.6%).

**Table 2:** Distribution of thrombosis in 194 patients with PVT

Site of thrombotic involvement^#^	No. of cases (%)
Portal vein only	145 (74.7)
Porto-mesenteric (SMV)	12 (6.2)
Porto-mesenteric (IMV)	0 (0)
Porto-venacaval	2 (1)
Porto-splenic	16 (8.2)
Porto-spleno-mesenteric (SMV)	19 (9.8)
Porto-spleno-mesenteric (IMV)	1 (0.5)

**^#^**Imaging modalities for the first diagnosis of PVT included ultrasonography (17%), Doppler ultrasonography (6.7%), CT (71.6%), MRI (1%), and MRV (3.6%).

### 3.2 Clinical presentations of patients with PVT

Table 3 shows the demographic data and clinical presentations of 194 PVT cases. These patients were predominantly males (126 cases, 65%) and aged older than 40 years (74.8%). The mean age at the time of admission was 50 (range, 0.2-82.9 years) and the affected male cases were older than the affected female cases (52.4 ± 15.7 SD vs 46.2 ± 20.8 SD, respectively). Only 6.7% of the cases were aged younger than 20 and 33% were older than 60. The most common clinical presentation in the younger patients, age<20 (84.6%) and age 20-39 (61.1%), was splenomegaly whereas in the older patients, age 40-60 (66.7%) and age>60 (76.6%), was abdominal distension/ascites. Abdominal distension/ascites was the most common clinical presentation in both males (43.8%) and females (25.3%).

**Table 3:** Clinical presentations of 194 patients with PVT

Sex (M:F)	126:68
Mean age ± SD (range)	50.2 ± 17.9 (0.2-82.9)
- Male	(n = 126) 52.4 ± 15.7 (1-80.8)
- Female	(n = 68) 46.2 ± 20.8 (0.2-82.9)
Age <20 (no. of cases, %)	13 (6.7)
Age 20-39 (no. of cases, %)	36 (18.6)
Age 40-60 (no. of cases, %)	81 (41.8)
Age >60 (no. of cases, %)	64 (33)
**Clinical presentations**	**No. of cases (%)**
**Asymptomatic**	2 (1)
**Abdominal distension/ascites**	134 (69.1)
**Abdominal pain**	98 (50.5)
**Jaundice**	70 (36.1)
**Fever**	26 (13.4)
**Splenomegaly**	106 (54.6)
**Hemorrhage**	35 (18)*
**Loss of appetite**	56 (28.9)
**Weight loss**	63 (32.5)
**Nausea**	32 (16.5)
**Vomiting**	25 (12.9)
**Diarrhea**	14 (7.2)

*hematemesis (10), melena (6), hematochezia (3), hematemesis and melena (11), hematemesis and hematochezia (3), melena and hematochezia (1), hematemesis, melena and hematochezia (1)

There were 144 PVT patients (105 males and 39 females) who were diagnosed with cancer or cirrhosis with a mean age at the time of diagnosis of 55.8 ± 14.3 (range, 1.9-82.9 years). Fifty PVT patients (21 males and 29 females) with a mean age of 34.2 ± 17.7 (range, 0.2-65.9 years) did not have detectable cancer and cirrhosis. There was no age difference between both genders. Seventy-eight percent of PVT patients with cancer or cirrhosis presented with abdominal distension/ascites followed by splenomegaly (50.7%), abdominal pain (47.2%), jaundice (47.9%), weight loss (39.6%), loss of appetite (35.4%), nausea (16.7%), hemorrhage (14.6%), fever (13.9%), vomiting (13.2%), and diarrhea (7.6%), and 1.4% of these patients were asymptomatic. Sixty-six percent of PVT patients without cancer and cirrhosis presented with splenomegaly followed by abdominal pain (60%), abdominal distension/ascites (44%), hemorrhage (28%), nausea (16%), vomiting (12%), weight loss (12%), fever (12%), loss of appetite (10%), diarrhea (6%), and jaundice (2%). None of the patients in the latter group was asymptomatic.

### 3.3 Risk factors associated with PVT

Risk factors for PVT could be established in the majority of cases (176 cases, 90.7%). Eighty-four cases (43.3%) had a single risk factor, 89 cases (45.9%) had two risk factors, and 3 cases (1.5%) had three risk factors. Table 4 summarizes the risk factors identified to be associated with PVT in this study cohort. The most common cause was cancer (126 cases, 65%) followed by cirrhosis (98 cases, 50.5%), abdominal intervention (15 cases, 7.7%), abdominal inflammation/infection (18 cases, 9.3%, and bone marrow disorders (9 cases, 4.6%). The most common type of cancer was hepatocellular carcinoma (HCC) (102/126, 81%) followed by cholangiocarcinoma (CCA) (16/126, 12.7%), pancreatic carcinoma (3/126, 2.4%), and liver metastasis (3/126, 2.4%). One case each of gall bladder carcinoma, hepatoblastoma, and ovarian carcinoma was also found. A variety of infections were found to be associated with PVT including pancreatitis (3), liver abscess (4), cholangitis (4), splenic abscess (1), cholecystitis (1), omphalitis (1), and abdominal tuberculosis (1).

**Table 4:** Risk factors associated with PVT

**Malignancy**	**126 (65)**
HCC (102), CCA (16), pancreatic CA (3), gallbladder CA (1), hepatoblastoma (1), ovarian CA (1), secondary malignant neoplasm of liver (2)	
Cirrhosis*	**98 (50.5)**
HBV (38), HCV (12), Alcohol (15), HBV+HCV (2), HBV+alcohol (8), HCV+alcohol (3), cryptogenic (19), autoimmune (1) [Child-Pugh Class: A (29), B (30), C (30), unknown (9)]	
**Abdominal intervention**	**15 (7.7)**
Umbilical catheterization (5), splenectomy (2), cholecystectomy (5), hepatectomy (1), percutaneous transhepatic biliary drainage (PTBD) (1), Whipple operation with revised hepatojejunostomy and T-tube (1)	
**Abdominal inflammation/infection**	**18 (9.3)**
Pancreatitis (3), liver abscess (4), cholangitis (4), splenic abscess (1), cholecystitis (1), omphalitis (1), abdominal tuberculosis (1), sepsis (3)	
**Bone marrow (BM) disorders**	**9 (4.6)**
Polycythemia vera (3), primary myelofibrosis (2), essential thrombocytosis (1), paroxysmal nocturnal hemoglobinuria (2), acute myeloid leukemia (1)	
**Hereditary coagulation defects**	**1 (0.5)**
Protein S deficiency (1)	
**Anti-phospholipid syndrome**	**1 (0.5)**
**Oral contraceptive pills**	**4 (2)**
Others**	**2 (1)**
**Idiopathic causes**	**18 (9.3)**

*Include cases with hepatocellular carcinoma (HCC) (79) and cholangiocarcionoma (CCA) (1)

** Klippel-Trénaunay syndrome (1), splenic lymphangiomatosis (1)

Risk factors are categorized based on the patients' age and gender as shown in Table 5. The most common risk factor in the patients aged <20 was abdominal interventions (3 umbilical catheterization and 1 hepatectomy) which was found in 30.8% of the cases. Almost half of the young cases had unidentifiable risk factors (46.2%). In contrast, cancer was the predominant risk factor in the patients aged 20-39 (38.9%), aged 40-60 (69.1%), and aged >60 (86%). More PVT male cases had cancer than female PVT cases (74.6% vs 47.1%). Cirrhosis was more frequently found in males than females (61.9% vs 29.4%). A higher proportion of females had unidentifiable risks than males (16.2% vs 5.6%, respectively).

**Table 5:** Risk factors for PVT according to age and gender

Risk factors*	All patients	Age range (years)				Gender	
		
		<20	20-39	40-60	>60	Male	Female
	
	N (%) 194 (100)	N (%) 13 (6.7)	N (%) 36 (18.6)	N (%) 81 (41.8)	N (%) 64 (33)	N (%) 126 (65)	N (%) 68 (35.1)
**Cirrhosis**	98 (50.5)	1 (7.7)	6 (16.7)	52 (64.2)	39 (60.9)	78 (61.9)	20 (29.4)
**Malignancy**	126 (65)	1 (7.7)	14 (38.9)	56 (69.1)	55 (86)	94 (74.6)	32 (47.1)
**Abdominal intervention**	15 (7.7)	4 (30.8)	3 (8.3)	6 (7.4)	2 (3.1)	6 (4.8)	9 (13.2)
**Abdominal inflammation or infection**	18 (9.3)	1 (7.7)	5 (13.9)	7 (8.6)	5 (7.8)	7 (5.6)	8 (11.8)
**BM disorders, protein S deficiency, and APS**	11 (5.7)	1 (7.7)	3 (8.3)	5 (6.2)	2 (3.1)	5 (4)	6 (8.8)
**Oral contraceptive pills**	4 (2)	0 (0)	4 (11.1)	0 (0)	0 (0)	0 (0)	4 (5.9)
**Others**	2 (1)	0 (0)	2 (5.6)	0 (0)	0 (0)	1 (0.8)	1 (1.5)
**Idiopathic**	18 (9.3)	6 (46.2)	8 (22.2)	3 (3.7)	1 (1.6)	7 (5.6)	11 (16.2)

N, number of cases; BM, bone marrow; APS, anti-phospholipid syndrome

*Some patients had more than one risk factor.

### 3.4 Incidence of HBV and HCV infection in patients with PVT

The frequency of HBV and HCV infections in patients with PVT according to the HCC/cirrhosis status, age, and gender is shown in Table 6. Increasing incidence of HBV infection was observed with increasing age. None of the young patients (aged <20) had HBV or HCV infections whereas about a third of older patients (aged >40) had HBV infection and less than 3% had HCV infection. More males with PVT were affected with HBV than females (39% vs 5.9%, respectively). PVT patients with HCC or cirrhosis (51/121, 42.1%), especially in patients aged >40 (44/145, 30.3%) had HBV infection as the most important risk factor. HCV infection (17/121, 14%) and HBV/HCV co-infection (2/121, 1.7%) were not as common as compared to HBV infection alone. Patients without cirrhosis or HCC had a very low frequency of HBV and HCV infections.

**Table 6:** Frequency of HBV and HCV infection in patients with PVT according to HCC/cirrhosis status, age, and gender

Risk factors*	PVT with HCC or cirrhosis	PVT without HCC and cirrhosis	Age (years)				Gender	
			
			<20	20-39	40-60	>60	Male	Female
	
	N (%)	N (%)	N (%)	N (%)	N (%)	N (%)	N (%)	N (%)
**HBV infection^a^**	51 (42.1)	2 (2.7)	0 (0)	9 (0.25)	27 (33.3)	17 (26.6)	49 (39)	4 (5.9)

**HCV infection^b^**	17 (14)	1 (1.4)	0 (0)	0 (0)	13 (16)	5 (7.8)	12 (9.5)	6 (8.8)

**HBV and HCV co-infection^c^**	2 (1.7)	0 (0)	0 (0)	0 (0)	2 (2.5)	0 (0)	2 (1.6)	0 (0)

*P value between age groups = ^a ^0.091, ^b ^0.022, ^c ^0.420

### 3.5 Distribution of thrombus in PVT patients with or without cirrhosis and cancer

Table 7 shows the branch distribution of thrombus according to age and gender. In the young patients aged < 20 and 20-39 (79.4%), the main branch was commonly affected whereas in the older patients, the right branch was slightly more affected than the left and the main branches. With respect to gender, the main and the right branches were commonly involved than the left branch in males (64.8% and 65.6%, respectively) whereas the main branch was slightly more affected than other branches in females (62.7%). The most common distribution of thrombus in the patients with cancer and cirrhosis was the right branch whereas in the patients with cirrhosis only was the main branch. For patients without cancer and cirrhosis, the thrombus was equally distributed in all branches.

**Table 7:** Distribution of thrombus in the portal vein branches based on age and gender^#^

	Total	Right Branch N (%) 112 (61.9)	Left branch N (%) 82 (45.3)	Main N (%) 116 (64.1)	Confluence N (%) 10 (5.5)
**Age (years)**					
<20	10	4 (40)	2 (20)	7 (70)	0 (0)
20-39	34	17 (50)	12 (35.3)	27 (79.4)	1 (2.9)
40-60	75	47 (62.7)	41 (54.7)	43 (57.3)	5 (6.7)
>60	62	44 (71)	27 (43.5)	39 (62.9)	4 (6.5)

**Gender**					
-Male	122	80 (65.6)	57 (46.7)	79 (64.8)	7 (5.7)
-Female	59	32 (54.2)	25 (42.4)	37 (62.7)	3 (5.2)

**PVT with CA or cirrhosis**					
-CA only -Cirrhosis only	44 15	32 (72.7) 9 (60)	17 (38.6) 5 (33.3)	26 (59.1) 13 (86.7)	1 (2.3) 0 (0)
-CA with cirrhosis	80	52 (65)	43 (53.8)	43 (53.8)	5 (6.3)
**PVT without CA and cirrhosis**					
-BM disorders and other risks	8	6 (75)	7 (87.5)	6 (75)	1 (12.5)
-Others	34	13 (38.2)	10 (29.4)	28 (82.4)	3 (8.8)

**^#^**181 cases had a complete description of involved branches by imaging studies

More patients in the cirrhosis only group had portomesenteric thrombus (SMV) (5/18) whereas almost half of the patients with bone marrow (BM) disorders, protein S deficiency, and anti-phospholipid syndrome (APS) had portosplenic thrombus (5/11). Two cases of portocaval thrombosis were caused by cancer (100%). Porto-spleno-mesenteric (SMV) thrombus was more commonly found in cases without cancer or cirrhosis (10/19, 52.6%).

## 4. Discussion

A variety of etiologic factors of PVT has previously been described including hereditary and acquired coagulation disorders, autoimmune diseases, abdominal infections/inflammations, abdominal intervention, transplantation, cirrhosis, and malignancies [11-24]. In this study, the predominant risk factor was HCC (52.5%), followed by liver cirrhosis without cancer (9.3%), and abdominal infections/inflammations (9.3%). Although HCC has been reported as a risk factor for PVT in the Caucasian patients [20], the prevalence was not as high as in the Asian cases. A high prevalence of HCC-associated PVT was noted in several series requiring therapeutic interventions in Chinese patients [14,23]. Cirrhosis without cancer was, in contrast, reported at a higher percentage as a cause of PVT than our cases. In a study of 172 PVT cases from the Netherlands, 15 cases of HCC and 37 cases of cirrhosis were reported, which represented 8.7% and 21.5% of the total cases, respectively [16]. A study from Sweden also reported that HCC and cirrhosis without cancer could be identified in 23% and 28% of the 254 PVT cases, respectively, although the exact etiologies of cirrhosis and HCC were not delineated in either study [1]. Our results, however, were as expected with respect to the natural history of cirrhosis and HCC and its relationship to thrombosis. Increasing incidence of PVT was observed in patients with liver decompensation and HCC when compared to patients with well-compensated cirrhosis [3,17,25].

The most common cause of cirrhosis in this patient cohort was HBV infection (38/98, 38.8%) which was much more prevalent than HCV infection (12/98, 12.2%). Alcohol was also the frequent cause of cirrhosis (15/98, 15.3%), and the combination of HBV, HCV and alcohol was found in 11 cases (11.2%). Patients without cirrhosis or HCC had a very low incidence of HBV or HCV infection. Our study is unique in that the majority of our PVT cases with underlying cirrhosis and HCC were associated with chronic HBV infection. HCV infection was not as prevalent as HBV infection alone as the cause of PVT which could be explained by the strikingly low incidence of HCV infection in the Southeast Asian population as compared to the Western populations [26,27]. Moreover, alcohol was the most common risk factor for HCC in a series reported from the United States [20]. Although HBV vaccination program has been implemented for all neonates in Thailand since 1992, the majority of our older generation is still not immunized and at least 4-8% of the population continues to be affected by the infection [26,28,29]. It is thus likely that HBV-associated cirrhosis/HCC will continue to be the major risk factor of PVT in the next few decades.

The risk factors of PVT appeared to vary according to age and gender. In young patients aged <20 years, umbilical catheterization was the main risk which was similar to the study of 31 children with PVT in Italy [30], whereas in older adults (age 40-60 and >60), malignancy was the major etiology (69.1% and 86%, respectively). Only 1 case of cancer, i.e. hepatoblastoma, was found in a young patient with PVT. Abdominal infection such as omphalitis which causes thrombophlebitis of the umbilical vein and eventually the portal system was reported in most pediatric series [21,31] but was found in only 1 case in this study (7.7%). Almost half of our young patients had unidentifiable risks (46.2%) which could be attributed to truly idiopathic causes or incomplete thrombophilic investigations. Although the main limitation of this study is its retrospective nature and a complete set of thrombophilic investigations might not have been performed in some of these cases in the last 10 years, it should be noted that hereditary thrombophilic disorders such as Factor V Leiden, prothrombin gene G20210A mutation, protein C deficiency, protein S deficiency, antithrombin deficiency, and methylenetetrahydrofolate reductase (MTFR) gene mutation are very rare in the Southeast Asian population [32,33]. A study of 61 patients with PVT from India reported only 1 case of Factor V Leiden and none with prothrombin gene G20210A mutation [34]. A prospective study should be performed to further explain these unidentifiable causes. Regarding the impact of gender, men were more commonly affected with cirrhosis and malignancies than women although both conditions remained the major risk factors in both genders.

Several risk factors identified in this population have rarely been described and are remarkably different from reports from the Western countries. Tuberculosis which is a prevalent infection in the Southeast Asia region was also found associated with PVT although abdominal tuberculosis is not a common manifestation [6]. In this study, a 32-year-old man who was previously diagnosed with abdominal tuberculosis came to our hospital because of hematemesis, melena, and splenomegaly. Endoscopic studies showed esophago-gastric varices with portal-hypertensive-gastropathy and CT scan of the whole abdomen revealed PVT. Two single cases of rare malformation syndromes such as Klippel-Trénaunay vascular malformation syndrome [9] and systemic lymphangiomatosis [10], which had been sporadically reported, were also discovered in our series. As for the first case, a 25-year-old woman came to the hospital because of epigastric pain radiating to midback associated with nausea and vomiting. The patient had splenomegaly and lymphadenopathy and CT study of the whole abdomen showed portal-spleno-mesenteric vein thrombosis with multiple cysts in the liver, pancreas, and spleen. A lymph node biopsy revealed lymphangioma; therefore this patient was diagnosed with systemic lymphangiomatosis with PVT which is the first case ever reported from the Southeast Asian population. The second case was a 29-year-old man with Klippel-Trénaunay syndrome who came to the hospital because of epigastric pain radiating to midback and was found to have an enlarged spleen on examination. He was subsequently found to have portal-spleno-mesenteric vein thrombosis by CT imaging. This case also represents the first case of Klippel-Trénaunay syndrome associated with PVT ever reported from the Southeast Asian region.

Bone marrow disorders such as paroxysmal nocturnal hemoglobinuria (PNH) and myeloproliferative neoplasms (MPN) including polycythemia vera (PV), chronic myeloid leukemia (CML), essential thrombocytosis (ET), and primary myelofibrosis (PMF) have been reported as presenting causes of abdominal venous thrombosis. In this study, we were able to identify only 2 cases of PNH-associated PVT among 497 medical charts extracted during a 10-year study period, supporting that venous thrombosis is rare in the PNH patients in Thailand [35] as contrast to the Western series [36]. Moreover, MPN which was found as a potential risk for PVT in the Western series necessitating a bone marrow study, endogenous erythroid colony (EEC) culture, and a *JAK2 *mutation analysis was infrequently identified in this cohort [24,37]. Only 3 cases of PV, 2 cases of PMF and 1 case of ET were discovered.

Other types of malignancies associated with PVT were also examined. In this study, secondary malignant tumors of the liver were identified in only 2 cases (1%). This frequency was much lower than the report by the Swedish autopsy series which found 111 PVT cases (44% of 254 cases) with secondary malignancy of the hepatobiliary region, and the major cause of which was pancreatic carcinoma (42%) [1]. The differences could be due to the fact that most autopsy series often revealed more occult cases of malignancies at the time of death than the clinical case reports performed while the patients still alive. Nevertheless, it could be concluded that metastatic cancer to the liver is an uncommon risk factor of PVT in this study. It is of interest to identify CCA as the second most common type of cancer associated with PVT in our population (16 cases, 8.2%). CCA is a rare cancer in the West but its prevalence is very high in Thailand and neighboring countries, the cause of which is mainly associated with *Opisthorchis viverrini *infection [38,39].

The clinical presentations of PVT patients in this study were typical of portal hypertension as a result of portal vein occlusion. A study from India performed in children younger than 14 years old showed that in the developing countries, 73% of extrahepatic portal hypertension were caused by PVT [8]. Splenomegaly which was the dominant clinical presentation in our patients without cancer and cirrhosis was consistent with the Danish's report [6]. However, most of our patients with cancer or cirrhosis had a slightly different presentation from those without cancer or cirrhosis since more cases in the first group presented with abdominal pain and ascites while the latter presented with splenomegaly. It could be argued that the abdominal pain symptom could be the result of HCC involvement of the liver, rather than PVT, however, it was too difficult to differentiate one another from the medical records.

It was of interest to find that CT was the most popular imaging modality for PVT diagnosis in this series. In general, Doppler ultrasonography is a preferred screening tool to diagnose thrombus in the portal system due to its lower costs, lack of need for intravenous contrast material injection, and ease of multiple follow-up examinations [40,41]. However, its sensitivity is operator-dependent which can vary from 60% to 100% [6,42-44]. CT has become widely available in Thailand for many years and it could be speculated that CT was performed in the majority of patients in this series (139 cases, 71.6%) because physicians felt that it was more accurate and more informative than ultrasonography to confirm the presence and extent of thrombus as well as to identify various abdominal etiologies associated with PVT [45]. MRI is another modality that can be used for PVT diagnosis with a high accuracy although its true sensitivities and specificities have never been reported for PVT diagnosis [46]. MRI was utilized only in a few patients in this series (9 cases, 4.6%) due to its limited availability and associated higher costs than CT and ultrasonography.

With respect to the sites of thrombus formation, it was of interest to find that the main branch was commonly affected in the younger patients whereas the right branch was slightly more affected than the left branch in the older patients. The reason why thrombi occur in different branches of the portal system in young and older patients remains unclear. However, with respect to the most common distribution of thrombus in patients with cancer and cirrhosis, our results were in agreement with a previous finding from Italy which revealed that among 701 patients with liver cirrhosis, the thrombus was found most commonly in the main portal trunk [3]. Moreover, a study from China also showed that the right branch was the most commonly affected branch in a series of 108 patients with advanced HCC [47].

## 5. Conclusion

One hundred and ninety-four cases of PVT were identified in a 10-year study period. The predominant identifiable risk factor was chronic HBV infection which is prevalent in this region and is strongly associated with cirrhosis and HCC. Interaction of host and environmental factors may play an important role in the pathogenesis of PVT in various ethnic populations residing in heterogeneous geographic regions. Future prospective studies such as gene association studies should be performed in order to identify the genetic risks and their modifying factors for PVT in these populations.

## Competing interests

The authors declare that they have no competing interests.

## Authors' contributions

KL was responsible for data collection and analysis and drafting of the manuscript. CUA was KL's major advisor who was responsible for the initiation and the execution of the project and the critical revision of the manuscript. Both authors read and approved the final manuscript.

## Pre-publication history

The pre-publication history for this paper can be accessed here:

http://www.biomedcentral.com/1471-230X/11/66/prepub
